# The Masking of Mourning: Social Disconnection After Bereavement and Its Role in Psychological Distress

**DOI:** 10.1177/2167702620902748

**Published:** 2020-05-11

**Authors:** Kirsten V. Smith, Jennifer Wild, Anke Ehlers

**Affiliations:** 1 Department of Experimental Psychology, University of Oxford; 2 Oxford Health NHS Foundation Trust, Oxford, England; 3 The Loss Foundation, London, England

**Keywords:** affective disorders, grief, posttraumatic stress disorder, social cognition, psychometrics

## Abstract

Social support has been shown to facilitate adaptation after bereavement in some studies but not others. A felt sense of social disconnection may act as a barrier to the utilization of social support, perhaps explaining these discrepancies. Factorial and psychometric validity of the Oxford Grief-Social Disconnection Scale (OG-SD) was tested in a bereaved sample (*N* = 676). A three-factor solution (negative interpretation of others’ reactions to grief expression, altered social self, and safety in solitude) fit the data best and demonstrated excellent psychometric validity. A second three-wave longitudinal sample (*N* = 275) recruited 0 to 6 months following loss and followed up 6 and 12 months later completed measures of prolonged grief disorder, posttraumatic stress disorder, depression, and the OG-SD at each time point. High levels of baseline social disconnection were associated with concurrently high psychological distress. The extent to which social disconnection declined over time predicted resolution of psychological distress.

Despite the widely held assumption that social support is important in facilitating grief adaptation, evidence for the role of social support in improving bereavement outcomes is inconsistent (Stroebe, Zech, Stroebe, & Abakoumkin, 2005). Although some studies have found that access to social support leads to better psychological outcomes following loss (Norris & Murrell, 1990; Okabayashi et al., 1997), others have not (Nolen-Hoeksema & Davis, 1999; Stroebe, Stroebe, & Abakoumkin, 1996). A recent study with parents bereaved by the 2011 Norwegian terrorist attacks found that those with high levels of social support at baseline were just as likely to experience prolonged grief disorder (PGD)^1^ 2 years later than parents with low or no social support (Wågø, Byrkjedal, Sinnes, Hystad, & Dyregrov, 2017). Conversely, Vanderwerker and Prigerson (2004) found social support in the first 6 months after loss to protect against the development of PGD, posttraumatic stress disorder, and depression 5 months later. The source of this discrepancy remains unclear. Perceived social support requires the bereaved individual to be willing and able to engage emotionally with their social network. In this article, we aim to introduce the concept of social disconnection as a potential barrier to the utilization of social support, present a measure with evidence for its factorial and psychometric validity, and provide empirical support for the role of social disconnection in maintaining psychological distress after bereavement.

The concept of social disconnection was derived from qualitative interviews with bereaved individuals with and without a diagnosis of PGD (Prigerson et al., 2009), which took place in the context of a study that explored the role of cognitive variables in adaptation following bereavement (Smith, 2018). Participants spoke about the negative consequences of emotional expression within a social context, which led them to conceal their grief. In particular, participants with PGD, more than those without, described feeling differently in the company of others since their loss, a felt sense that led to avoidance of social situations or a significant reduction in their ability to tolerate social situations for prolonged periods (Smith, Rankin, & Ehlers, 2019). This felt sense appeared to be driven by negative interpretations of other people’s observed or anticipated reactions to grief expressions and was accompanied by concerns about being authentic in one’s own grief when in the company of others as well as a wider sense of a changed self in social situations and alienation from others.

Catastrophic interpretations of emotional and bodily sensations have been implicated in the development and maintenance of several psychological problems (grief: Boelen, van den Bout, & van den Hout, 2010; panic disorder: Clark, 1986; social anxiety disorder: Clark & Wells, 1995; PTSD: Ehlers & Clark, 2000; obsessions: Rachman, 1997; depression: Treynor, Gonzalez, & Nolen-Hoeksema, 2003). Previous research has referred to anticipated negative consequences of grief for the individuals, such as, “If I allow my feelings to come I will lose control.” Interpretations of the impact of one’s behavior and appearance on other people have been described within the social anxiety literature. People with social anxiety disorder have “a strong desire to convey a particular favorable impression of oneself to others and marked insecurity about one’s ability to do so” (Clark & Wells, 1995, p. 69). Remarkably, people with social anxiety disorder based their interpretation that they were coming across badly from internal evidence, emotions (feeling anxious) and bodily sensations (feeling warm as a sign of blushing). The sense of social disconnection we observed in people with PGD appears related to social anxiety in that bereaved individuals fear being overwhelmed by their grief in a social setting and engage in considerable effort to maintain their composed presence (Smith, 2018). However, the interpretations of a changed self in social situations and associated felt sense of social disconnection can arise in individuals who may have previously never experienced socially dependent fears and are specific to grief in that they include judgments about their social network’s readiness to tolerate and empathize with their expressed grief as well as judgments about their own desire to share their grief.

Items for the Oxford Grief-Social Disconnection Scale (OG-SD) were generated from interview transcripts in which bereaved participants described changes in social processing since their loss (Smith, 2018). Our aim was to examine the factorial and psychometric validity of the OG-SD and investigate its utility in predicting psychological distress after bereavement over time. Using a large community sample, we built the factor model, using exploratory factor analysis, on 50% of the sample and tested it, using confirmatory factor analysis, on the remaining 50%. In a three-wave longitudinal sample recruited in the first months after loss and followed up 6 and 12 months later, we investigated whether social disconnection predicted higher psychological distress at baseline and during follow-up. Because bereaved people may exhibit a broad range of symptoms and social disconnection may play a role in many of them, psychological distress was operationalized as a latent factor composed of symptoms of PGD, posttraumatic stress disorder (PTSD), and depression. We aimed to address incremental validity for the new social disconnection scale in two ways. First, we tested whether the OG-SD explained additional variance in the intercept and slope of the latent psychological distress factor compared with a composite of two symptom items that are closest conceptually to social disconnection; “feeling distant or cut off from others,” which is part of Cluster D in the diagnosis of PTSD (American Psychiatric Association, 2013), and “finding it hard to trust others since the loss,” part of Criterion D in the diagnosis of PGD (Prigerson & Maciejewski, 2008). Second, we tested whether the OG-SD explained variance in impairment in social and occupational functioning over and above that explained by symptom measures of PGD, PTSD, and depression; we used the Work and Social Adjustment Scale (WSAS), a measure commonly used to assess impairment that results from physical and mental health problems (Mundt, Marks, Shear, & Greist, 2002).

## Method

### Participants and procedure

The results presented use three separate samples of bereaved individuals recruited through bereavement charity mailing lists, via social media advertisements, and from the Google content network. The first cross-sectional sample included 676 adults (mean age = 49.22 years, *SD* = 12.52; 81.5% women) bereaved at least 6 months previously (mean months since loss = 56.81, *SD* = 79.79); 36.1% lost a partner, 28.3% lost a parent, 21.0% lost a child, 6.5% lost a sibling, and 8.2% lost another relative or close nonrelative. Nineteen percent lost a loved one via violent^2^ means.

The second sample of 50 individuals was used to investigate test–retest reliability of the OG-SD measures. Participants completed the measures twice with a 1-week gap. Their mean age was 51.46 years (*SD* = 14.54), and 84.0% were women; 42% had lost a parent, 28% had lost a partner, 22% had lost a child, and 8% had lost another relative or close nonrelative. Individuals were bereaved on average 23.74 months (*SD* = 48.44) before the study, and 26% lost a loved through a violent death.

The third longitudinal sample consisted of 275 adults (mean age = 46.43 years, *SD* = 13.24; 79% women) recruited between a few weeks and 6 months after bereavement (Time Point 1: *M* = 2.94 months, *SD* = 2.01, range = 0–8 months) and then followed up 6 months (Time Point 2: *M* = 9.10 months, *SD* = 2.23, range = 6–16 months) and 12 months later (Time Point 3: *M* = 14.95 months, *SD* = 2.08, range = 12–21 months). In this sample, 38.2% lost a parent, 30.2% lost a partner, 8.7% lost a child, 5.8% lost a sibling, and 17.1% lost another relative or close nonrelative. Nine percent lost a loved one via violent means.

Participants completed symptom measures and the OG-SD online in accordance with ethical guidelines (Smith, Thew, & Graham, 2018). Participants were compensated for their time. Informed consent was obtained from participants electronically, and the studies were approved by the University of Oxford Medical Sciences Inter-Divisional Research Ethics Committee (MS-IDREC-C1-2015-230; MS-IDREC-C1-2015-231).

### Measures

#### Cognitive measures

##### The Oxford Grief Social Disconnection Scale

The OG-SD includes 15 items developed from interviews. Items reflect how bereaved individuals feel about sharing their grief-related thoughts and feelings with others (e.g., “Others will not be able to manage if I tell them how I feel about the loss”), their sense of inauthenticity (e.g., “When I am with other people, I feel I am putting on a performance”), a preference for solitude that arises from these difficulties (e.g., “It is better to be by myself than to show others how I am really feeling”), and a perceived change in the social self (“I don’t fit in socially the way I used to”). Participants were asked to rate the extent to which they agreed with each statement on a 7-point scale (1 = *totally disagree*, 7 = *totally agree*).

##### The Oxford Grief Coping Strategies Scale–Avoidance subscale

The Oxford Grief Coping Strategies Scale (OG-CS) is a 23-item questionnaire that asks participants on a 5-point scale (1 = *never*, 5 = *always*) to indicate how often they used particular strategies to cope with their loss. Items pertain to four content domains: avoidance, proximity seeking, grief rumination, and injustice rumination. The six-item avoidance subscale measures the extent to which bereaved individuals avoid specific situations (e.g., “I avoid places we went together”), activities (e.g., “I avoid watching television programmes that remind me of [—] or death in general”), and experiences (e.g., “I make an effort to hold back my feelings”). Internal consistency was acceptable in the cross-sectional sample (*N* = 676, ω = .79) and good in the longitudinal sample (*N* = 275, ω = .87)

#### Symptom measures

##### Prolonged Grief Disorder Inventory

The Prolonged Grief Disorder Inventory (PG-13; Prigerson & Maciejewski, 2008) assesses the prevalence and severity of PGD symptoms (e.g., yearning for the deceased, feelings of emotional numbness/detachment from others, feeling that a part of oneself died along with the deceased). The PG-13 is a subset of 13 items from the Inventory of Complicated Grief (Prigerson et al., 1995). A continuous score can be derived using the sum of the score of each of the 11 grief symptoms and ranges from 11 to 55. Internal consistency was good in the cross-sectional sample (*N* = 676, α = .91), test–retest sample (*N* = 50, α = .89), and longitudinal sample (*N* = 275, α = .89).

##### Posttraumatic Stress Disorder Checklist for DSM–5

The Posttraumatic Stress Disorder Checklist for *DSM*–5 (PCL-5; Weathers et al., 2013) is a self-report instrument assessing distress associated with the 20 symptoms of PTSD in the fifth edition of the *Diagnostic and Statistical Manual of Mental Disorders* (*DSM–5*; American Psychiatric Association, 2013) over the past month. Items were rated on a 5-point scale (0 = *not at all*, 4 = *extremely*). A cutoff score of 33 has been recommended for a probable PTSD diagnosis. Internal consistency was excellent in all samples (cross-sectional α = .94, test–retest α = .94, longitudinal α = .94).

##### Patient Health Questionnaire

The Patient Health Questionnaire (PHQ-9; Kroencke, Spitzer, & Williams, 2001) is a self-report measure based on criteria for major depressive disorder from the fourth edition, text revision of the *DSM* (*DSM–IV–TR*; American Psychiatric Association, 2000). It mirrors the nine major depressive symptoms in the past 2 weeks. Each item is scored on a 4-point scale (0 = *not at all*, 3 = *nearly every day*). A cutoff score of 10 has been recommended for a probable diagnosis of depression. Internal consistency was excellent in all samples (cross-sectional α = .92, test–retest α = .92, longitudinal α = .91).

#### Social and occupational functioning

The Work and Social Adjustment Scale (WSAS; Mundt et al., 2002) assesses impairment of functioning in five dimensions (work, home management, social leisure activities, private leisure activities, and family and relationships) on a nine-item scale (0 = *not at all*, 8 = *very severely*). It was assessed at Time Points 2 and 3 for the longitudinal sample. Participants were asked to rate grief-related impairment when completing the measure.

### Data analyses

#### Preparation of the data

Data were processed using MATLAB (The MathWorks, Natick, MA) to identify “straight lining” (i.e., participants who repeatedly select the same response throughout questionnaires). This process is important in determining data quality of measures collected online in which participants may be financially motivated to take part and thus not provide true information (Tourangeau et al., 2018; Zhang & Conrad, 2014). Participants were flagged if they chose the same response on more than 80% of each questionnaire’s items. Participants with multiple flags were then examined on a case-by-case basis. Time taken to complete the measures is recorded automatically by Qualtrics survey software (Qualtrics, Provo, UT) and used to further corroborate if straight-lining participants had provided unreliable data. These data checks revealed that no participants had repeated flags and were quick to complete the measures. Therefore, no data were excluded.

#### Factorial validity

To cross-validate the measurement model developed, the data were subject to a 50% random split (Osborne & Fitzpatrick, 2012). Exploratory factor analysis (EFA) was used to build the measurement model on one half of the data, and the measurement model was tested on the other half using confirmatory factor analysis (CFA). There is debate within the literature about the suitability of using parametric statistical procedures with Likert scales (Carifio & Perla, 2007; Jamieson, 2004). It has been suggested that Likert scales with 5 to 7 response points (7 being better) perform well on parametric techniques such as *F* ratio and thus can be treated as continuous data (Glass, Peckham, & Sanders, 1972; B. O. Muthén & Kaplan, 1985; Rhemtulla, Brosseau-Liard, & Savalei, 2012). However, community samples measuring rate or features of mental health problems are often skewed (Baker et al., 2016; Boelen & Lensvelt-Mulders, 2005). Therefore, we adopted a maximum likelihood robust (MLR) estimation using Mplus (Version 8; L. K. Muthén & Muthén, 2017), which is robust in the presence of nonnormality.

Because scale factors were expected to correlate, geomin oblique rotation was used (L. K. Muthén & Muthén, 2007). First, a χ^2^ goodness-of-fit test in which the χ^2^:*df* ratio was smaller than 3:1 was regarded as acceptable. Second, comparative fit index (CFI) values of ≥ .90 or ≥ .95 were considered acceptable or good, respectively. Third, root mean square error of approximation (RMSEA) values of ≤ .10 or ≤ .06 were considered acceptable or good, respectively (Hu & Bentler, 1999; MacCallum, Browne, & Sugawara, 1996). Factor determinacy was assessed by factor loadings greater than .35, and items with comparable cross-loadings were placed with the factor that made the most conceptual sense. Items without strong factor loadings were ultimately placed on the factor on which they loaded most strongly. Modification indices (MI) (i.e., the improvement in model’s χ^2^ by freeing the residual variance correlation between two items) were considered only when large (> 10) and the suggested correlated errors fit with the conceptual interpretation (Brown, 2014). To support the use of a total score on the OG-SD, a higher-order factor was fit to the data after an adequate confirmatory factor model had been established (Brown, 2014).

#### Psychometric validation

Internal consistency was assessed by Cronbach’s α. Criterion and convergent validity for the total scale was determined using correlations with measures of psychopathology (i.e., PGD, PTSD, and depression) and behavioral avoidance (i.e., OG-CS–Avoidance subscale). An average variance extracted (AVE) score was also calculated for each factor to determine the average variance in the latent factor that is accounted for by its items (Fornell & Larcker, 1981). A score of .50 or higher confirms factorial convergent validity (Hair, Ringle, & Sarstedt, 2011). Discriminant validity of factors was determined if the AVE from a latent construct was larger than the highest squared interconstruct correlation (Henseler, Ringle, & Sarstedt, 2015). This metric indicates whether each questionnaire subscale is sufficiently different from the other subscales of the measure. The stability of the total scale and subscales over time was measured using the test–retest reliability sample. A correlation greater than .70 between two time points a week apart was used to indicate acceptable retest reliability.

#### Structural equation modeling

**Fig. 1.**
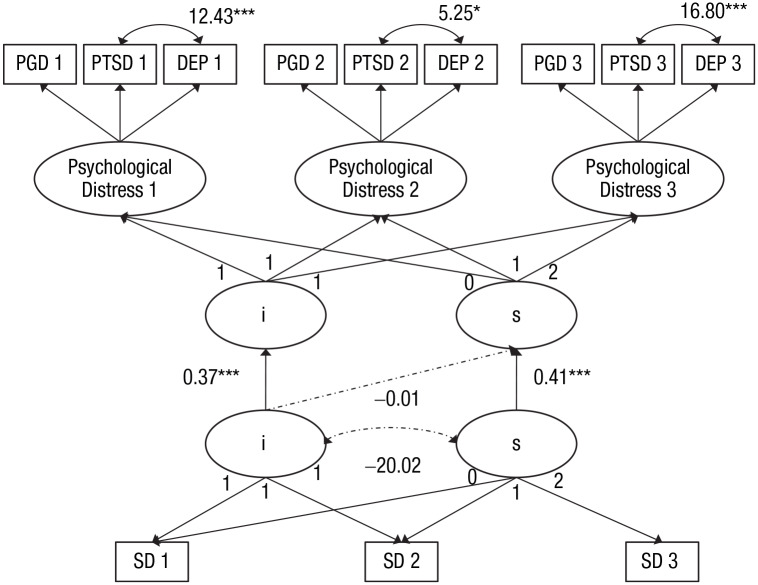
Structural equation model of the curve of factors model of psychological distress and the latent growth curve of social disconnection. Unstandardized coefficients are shown. PGD = prolonged grief disorder score (Prolonged Grief Disorder Inventory) with social symptom composite item removed; PTSD = posttraumatic stress disorder score (Posttraumatic Stress Disorder Checklist for *DSM–5*) with social symptom composite item removed; DEP = depression score (Patient Health Questionnaire); SD = social disconnection score (Oxford Grief-Social Disconnection Scale); i = intercept; s = slope. Scalar invariance was specified using the marker variable approach. The residual variances among the same indicators over time were correlated but are not shown in the figure for simplicity. Solid lines indicate statistically significant paths. Broken lines indicate nonsignificant paths. χ^2^(51) = 160.28, χ^2^:*df* = 3.14, comparative fit index = .96, Tucker-Lewis index = .95, root mean square error of approximation = 0.088, standardized root mean square residual = .05. Asterisks indicate significance of parameters (*p < .05, ***p < .001).

To determine the role of social disconnection on psychological distress, two models were built separately using Mplus (Version 8; L. K. Muthén & Muthén, 2017). The first model fit a latent growth curve (LGC) using the total score of the social disconnection scale. LGC modeling produces an intercept and a slope factor score for each individual. The second model was built in three steps: A longitudinal CFA (LCFA) was conducted in which the total scores for the PGD, PTSD, and depression scales at Times Points 1, 2, and 3 load onto latent factors of psychological distress at each time point. To minimize dependency (i.e., overlap with the OG-SD scale), one symptom was removed from the PGD sum score (“finding it hard to trust others since the loss”) and one from the PTSD sum score (“feeling distant or cut off from others”). This model was then subject to invariance testing to assess its suitability in measuring psychological distress over time.^3^ Next, growth terms (intercept and slope) were added to the LCFA to produce a curve of factors model (CUFFS). To minimize the bias associated with attrition and missing data, we used the full information maximum likelihood approach implemented in Mplus to estimate missing data. The following fit indices were used to determine adequate fit: χ^2^:*df* ratio < 3:1, CFI > .90, TLI > .90, standardized root mean squared residual (SRMR) < .08 (Hu & Bentler, 1999; Wickrama, Lee, O’Neal, & Lorenz, 2016), and RMSEA < 0.10 (Browne & Cudeck, 1992; MacCallum et al., 1996). Once two well-fitting solutions were modeled, the intercept and slope of psychological distress were then regressed on the intercept and slope of the OG-SD to observe whether perception of social disconnection in the first months of loss predicted initial psychological distress and whether change in social disconnection predicted psychological distress over time (see Fig. 1).

#### Incremental validity

##### Psychological distress

We examined^4^ whether the total score of the OG-SD at baseline explained additional variance in the intercept and slope of the psychological distress compared with the composite of the social symptoms “feeling distant or cut off from others” and “finding it hard to trust others” at baseline. Factor scores of the CUFFS model of psychological distress were saved and reimported into the data to stabilize the model. Two hierarchical regression analyses were run using the intercept and slope of psychological distress as the dependent variable. The social symptoms composite at baseline was entered in the first step, and the OG-SD at baseline was entered at the second step. We report the change in explained variance (*R*^2^), an indicator of the additional variance accounted for by the OG-SD.

##### Social and occupational functioning

The WSAS was assessed at Time Points 2 and 3 in the longitudinal sample.^5^ Six hierarchical regression analyses were run in the prediction of the WSAS, 2 time points × 3 symptom scales (either PGD, PTSD, or depression). The symptom scales were entered in the first step, and the OG-SD was entered at the second step. Including the full PG-13 and PCL-5 scales, without removing any variables that were likely to overlap with the OG-SD, ensured that only variance unique to the OG-SD was measured at the second step. We report the change in explained variance (*R*^2^) as an indicator of the additional predictive utility of the OG-SD. To account for these multiple comparisons, Bonferroni adjustment set the significance level for each univariate model to *p* < .008 (.05/6).

## Results

### Exploratory factor analyses

All 15 social disconnection items were subject to exploratory factor analyses on the EFA sample (*N* = 348). Inspecting eigenvalues greater than 1 was suggestive of a three-factor structure (8.10, 1.24, 1.12). Examination of the scree plot could have supported a two-, three-, or four-factor solution. The two-factor model indicated a borderline acceptable fit for CFI (.90) and RMSEA (0.098) but showed a poor fit for χ^2^, χ^2^(76) = 305.28, χ^2^:*df* = 4.02. The three-factor model indicated a good fit for CFI (.95) and an acceptable fit for RMSEA (0.073) and χ^2^, χ^2^(63) = 168.87, χ^2^:*df* = 2.68. The four-factor model did not converge. The two-factor solution had two strong cross-loadings (i.e., “When I am with other people, I feel I am putting on a performance” and “It is easier to be alone than to have to pretend to feel ok”). The three-factor solution had the same strong cross-loadings and one weak loading item (< .35; i.e., “Others will not be able to manage if I tell them how I feel about the loss”). Inspection of the modification indices suggested that a correlated error should be added between “If I show my real feelings other people will think I am not normal” and “Others would judge me if I were to speak openly about my grief” (MI = 43.86). This suggested correlated error is likely due to the fact that these items appear on a different page of the study questionnaire and represent a substantively irrelevant method effect (Brown, 2014). The three-factor solution was considered optimal given the fit statistics.

The social disconnection items and standardized factor loadings are presented in Table 1. Factors were labeled Negative Interpretation of Others’ Reactions to Grief Expression, Altered Social Self, and Safety in Solitude.

**Table 1.** Factor Analyses of the Social Disconnection Scale

		Factors					
		1		2		3	
Social disconnection items		EFA	CFA	EFA	CFA	EFA	CFA
1	If I show my real feelings other people will think I am not normal.	.66	.62	—	—	—	—
2	Others would judge me if I were to speak openly about my grief.	.97	.64	—	—	—	—
3	Others will not be able to manage if I tell them how I feel about the loss.	.32	.84	—	—	—	—
4	The company of others makes me feel uncomfortable.	—	—	.72	.76	—	—
5	I need to be able to leave social situations when I want or I will break down.	—	—	.64	.75	—	—
6	I can’t be myself around other people the way I used to.	—	—	.72	.84	—	—
7	I feel alien to those around me.	—	—	.83	.79	—	—
8	I don’t fit in socially the way I used to.	—	—	.89	.83	—	—
9	I find it draining to be around other people.	—	—	.80	.81	—	—
10	When I am around other people, it feels like I am ruining their enjoyment.	—	—	.76	.74	—	—
11	When I am with other people, I feel I am putting on a performance.	—	—	.58	.81	—	—
12	It is better to be by myself than to show others how I am really feeling.	—	—	—	—	.54	.79
13	I can only let my true feelings show when I am on my own.	—	—	—	—	.84	.84
14	I can only be myself when I am on my own.	—	—	—	—	.71	.85
15	It is easier to be alone than to have to pretend to feel ok.	—	—	—	—	.48	.78
Correlations matrix of OG-SD factors							
	Factor 1	—	—				
	Factor 2	.54	.82	—	—		
	Factor 3	.42	.74	.60	.79	—	—
Higher-order – Social disconnection subscale loadings			.87		.85		.94

Note: EFA *N* = 348. CFA *N* = 328). Factors were labeled as follows: 1. Negative Interpretation of Others’ Reactions to Grief Expression, 2. Altered Social Self, and 3. Safety in Solitude. All factor loadings significant to *p* < .05. EFA = exploratory factor analysis; CFA = confirmatory factor analysis; OG-SD = Oxford Grief-Social Disconnection Scale.

### Confirmatory factor analyses

The CFA assessed the fit of chosen three-factor solution with one correlated error using the CFA sample (*N* = 328). The fit statistics for the three-factor model indicated acceptable fit, CFI = .94, RMSEA = 0.075, χ^2^(86) = 230.13, χ^2^:*df* = 2.68. A χ^2^ difference test using the Satorra-Bentler correction for MLR estimations was significant in a comparison with the two-factor model and the three-factor model, which indicates that three factors fit the data better than two, χ^2^(1) = 82.51, *p* < .001). If a first-order model has three factors, a solution that specifies a single higher-order factor will be just-identified (i.e., the higher-order solution will produce the same goodness of fit as the first-order model; Brown, 2014). In these circumstances, standardized factor loadings are examined to determine the magnitude and statistical significance of the higher-order factor loadings. All three-factor loadings were statistically significant (*p* < .001), which supports a sum score of the social disconnection items as well as subscale factor scores. Table 1 summarizes the standardized factor loadings for the three-factor and higher-order factor solutions and the interfactor correlation matrix.

### Psychometric validation

Validity and reliability metrics are reported in Table 2. Internal consistency for the total OG-SD and its subscales were good or excellent. Test–retest reliability for the total OG-SD was good, as was the reliability of the Altered Social Self subscale. However, both Negative Interpretation of Others’ Reactions to Grief Expression and Safety in Solitude had only moderate retest-reliability, which indicates that these scales may be less stable over time. Correlations between the total score of the OG-SD, its subscales, and symptom measures of PGD, PTSD, and depression were all moderate or strong and significant, which confirms criterion validity. Correlations with avoidant coping strategies were also moderate or strong and significant.

**Table 2.** Psychometric Validity of Total Social Disconnection Scale (OG-SD) and Latent Factors

			Factors		
Reliability/validity	Measure	Total scale	1	2	3
Reliability	Cronbach’s α	.94	.80	.93	.89
	Test–retest *r*	.80***	.58***	.81***	.69***
Validity criterion	PGD *r*	.62***	.45***	.62***	.52***
	PTSD *r*	.67***	.51***	.66***	.55***
	Depression *r*	.63***	.44***	.64***	.52***
	Avoidance (OG-CS) *r*	.64***	.54***	.62***	.51***
Convergent	AVE		.50	.63	.67
Discriminate	Largest interconstruct *r*^2^		.66	.66	.63

Note: Factors were labeled as follows: 1. Negative Interpretation of Others’ Reactions to Grief Expression, 2. Altered Social Self, and 3. Safety in Solitude. Test–retest reliability confirmed if *r* > .70. Convergent validity of factors confirmed if AVE > .5. Factorial discriminant validity confirmed if AVE > largest interconstruct *r*^2^. *r =* correlation; AVE = average variance extracted; OG-SD = Oxford Grief-Social Disconnection Scale; OG-CS, Oxford Grief Coping Strategies Scale; PGD = prolonged grief disorder; PTSD = posttraumatic stress disorder.

*** *p* < .001.

Although factorial convergent validity was confirmed for the three subscales, factorial discriminant validity was confirmed only for the Safety in Solitude subscale. The Negative Interpretation of Others’ Reactions to Grief Expression and the Altered Social Self subscales failed to meet factorial discriminant validity because of their high correlation (*r* = .82). The squared correlation of these subscales (*r*^2^ = .66) was larger than each subscale’s AVE score. However, as already described, a three-factor model fit the data significantly better than the two-factor model, which suggests that these items should be on separate factors.

### Longitudinal analyses

#### Descriptive statistics

Descriptive statistics of the OG-SD and symptom variables PGD, PTSD, and depression at Time Points 1, 2, and 3 are shown in Table 3.

**Table 3.** Descriptive Statistics of Social Disconnection, PGD, PTSD, and Depression

	Time Point		
Measure	1	2	3
OG-SD	63.01 (23.03)	60.08 (23.88)	57.93 (24.18)
PGD	34.63 (10.15)	29.34 (10.15)	26.33 (10.32)
PTSD	34.04 (19.04)	26.37 (17.32)	23.17 (17.78)
Depression	12.60 (7.60)	9.33 (6.73)	8.68 (7.17)

Note: Symptom scale means and standard deviations (in parentheses) are reported in full before removal of the social symptoms composite items. OG-SD = Oxford-Grief Social Disconnection Scale; PGD = Prolonged Grief Disorder Scale (Prolonged Grief Disorder Inventory); PTSD = posttraumatic stress disorder (Posttraumatic Stress Disorder Checklist for *DSM–5*); depression = Patient Health Questionnaire.

#### Growth curve modeling

The unconstrained longitudinal confirmatory factor analysis of psychological distress, with social symptoms composite items removed, was a good fit for CFI (.98), TLI (.95), and SRMR (.07) but was above threshold for χ^2^—χ^2^(15) = 57.21, χ^2^:*df* = 3.41—and RMSEA (0.10). Modification indices suggested freeing the correlated error between PTSD and depression at Time Points 2 and 3. Given the significant overlap in symptoms (i.e., five of the nine symptoms of depression are also symptoms of PTSD; Flory & Yehuda, 2015) and high levels of comorbidity between PTSD and depression (Kessler, Sonnega, Bromet, Hughes, & Nelson, 1995), this was deemed an appropriate modification to the model because it acknowledges that some of the shared variance between depression and PTSD is attributable to the similar items measuring both constructs. Therefore, three correlated errors, between PTSD and depression at each time point, were added to the model. The LCFA of psychological distress with three correlated errors was an excellent fit to the data, χ^2^(12) = 16.17, χ^2^:*df* = 1.35, CFI = 1.00, TLI = .99, SRMR = .04, RMSEA = 0.02. Equality constraints of metric and scalar measurement invariance did not substantially reduce model fit, which confirmed that the model was stable across time (see the Supplemental Material available online). Growth terms (intercept and slope) were then added to the LCFA to produce a (CUFFS) model. The CUFFS of psychological distress with scalar invariance constraints and three correlated errors was an excellent fit to the data according to CFI (.97), TLI (.96), and SRMR (.06); an acceptable fit according to RMSEA (0.092); but was above the cutoff for χ^2^—χ^2^(22) = 72.74, χ^2^:*df* = 3.30. Given four out of five fit statistics were acceptable or excellent, overall fit was deemed adequate to proceed.

A single linear growth curve of the OG-SD scores over time was an excellent fit to the data on all metrics, χ^2^(3) = 2.30, χ^2^:*df* = .77, CFI = 1.00, TLI = 1.00, SRMR = .05, RMSEA = 0.00.

When combined into one model, with regression paths specified between the intercept and slope of psychological distress and the intercept and slope of social disconnection, the LGC of social disconnection and the CUFFS of psychological distress estimated nonsignificant negative variances of the slope of psychological distress and the latent factor of psychological distress at Time Point 3. Thus, the variances of these paths were fixed to 0 (Wickrama et al., 2016). The constrained model was not statistically different from the initial estimated model, Δχ^2^(3) = 0.97, *p* = .81. Thus, we proceeded with the constrained model, which was also an acceptable fit to the data on all metrics (CFI = .96, TLI = .95, RMSEA = 0.088, SRMR = .05), except χ^2^(51) = 160.28, χ^2^:*df* = 3.14, which was above the cutoff. Overall fit was deemed acceptable for interpretation. High levels of perceived social disconnection at baseline predicted higher levels of psychological distress at baseline (*b* = 0.37, *SE* = 0.02, *p* < .001). The regression between the intercept of social disconnection and the slope of psychological distress was not significant (*b* = −0.01, *SE* = 0.01, *p* = .38). However, the regression between the slope of social disconnection and the slope of psychological distress was highly significant (*b* = 0.41, *SE* = 0.05, *p* < .001), which suggested that the extent to which social disconnection reduced over time was associated with faster resolution of psychological distress (Fig. 1).

#### Incremental validity

##### Psychological distress

Results of the regression analyses showed that the OG-SD (Step 2) explained additional variance in the intercept of psychological distress over and above what could be explained by the social symptoms composite (Step 1). The social symptoms composite explained a significant amount of variance in the first step, *F*(1, 267) = 261.03, *p* < .001, *R*^2^ = .49. In the second step, the OG-SD increased the variance explained in the intercept of psychological distress by 12%, a change that was highly significant, Δ*F*(1, 266) = 86.45, *p* < .001; Δ*R*^2^ = .12.

For the slope of psychological distress, in Step 1, the social symptoms composite did not significantly explain variance in change in psychological distress, *F*(1, 267) = 2.76, *p* = .10, *R*^2^ = .01, and in line with the structural equation modeling, neither did the OG-SD at baseline, Δ*F*(1, 266) = 0.02, *p* = .88, Δ*R*^2^ = .00.

##### Social and occupational functioning

A series of hierarchical regressions using the WSAS as the dependent variable at Time Points 2 and 3; the symptom scales PGD, PTSD, and depression at the first step; and the OG-SD at the second were conducted.^6^ The OG-SD explained between 3% and 11% additional variance in the WSAS compared with the symptom scales at each time point. All changes were highly significant after Bonferroni correction (*p* < .001).

## Discussion

In our study, we explored the factorial and psychometric properties of the Oxford Grief-Social Disconnection Scale—a measure that was developed from interviews with bereaved individuals to reflect social cognitions relevant to the development and maintenance of psychological distress following bereavement. Exploratory and confirmatory factor analyses supported a three-factor solution: Negative Interpretations of Others’ Reactions to Grief Expression (e.g., “Others will not be able to manage if I tell them how I feel about the loss”), Altered Social Self (e.g., “I don’t fit in socially the way I used to”), and Safety in Solitude, which described a sense that being alone provided safety by allowing authenticity of the self and of one’s grief (e.g., “I can only be myself when I am on my own”). The OG-SD demonstrated acceptable to excellent internal consistency, convergent validity, and test–retest reliability. Test–retest reliability was good for the Altered Social Self subscale but was only moderate for the other two subscales. Discriminant validity was also confirmed in the Safety in Solitude subscale but not for the Altered Social Self and the Negative Interpretation of Others’ Reactions to Grief Expression subscales because of their high correlation. Model comparison confirmed that three factors fit the data better than two, which indicated the items on these scales were better represented on separate factors. Their high correlation may indicate two aspects of the same problem. A felt sense of an altered social self may be driven by the belief that others will not be able to understand or tolerate a person’s grief.

The role of social disconnection in predicting psychological distress was supported with latent growth curve modeling that showed higher levels of social disconnection to be associated with higher psychological distress in the first 6 months of loss. In addition, greater decline in social disconnection over time was associated with greater reductions in psychological distress.

The OG-SD also demonstrated incremental validity by explaining additional variance in baseline psychological distress compared with the social symptoms composite made up of conceptually similar items from the PGD and PTSD scales (“feeling distant or cut off from others” and “find it hard to trust others since the loss”) and in social and occupational functioning compared with measures of mental health problems after loss (PGD, PTSD, and depression). These results suggest that the chosen items of social disconnection have additional predictive power in understanding psychological distress and impairment compared with symptom items related to social relationships and the total scales measuring diagnostic criteria.

The OG-SD describes negative interpretations of social experiences that arise when grieving and an associated sense of disconnection of the self from others. While holding and managing the difficult emotions associated with grief, individuals engage in emotional suppression in the company of others for fear that their grief would be unacceptable to others or would reflect negatively on them. As a result, individuals “perform” emotional expressions that are incongruent to those held internally, and the associated discomfort resulting from this process motivates a preference for solitude, reduced social engagement, or both because of the cognitive and emotional demands necessary to emotionally suppress their grief. The repeated process of grief concealment may lead to an altered sense of self in social situations. Previous research has shown that in high-intensity emotion situations, people tend to prefer disengagement strategies, such as distraction, over engagement strategies, such as reappraisal (i.e., changing what is thought about a situation to decrease its emotional impact; Gross, 2002; Sheppes et al., 2014). This concept of an altered sense of self may best reflect those individuals for whom disengagement has become the chosen emotion regulation strategy.

The cognitive and social costs of expressive dissonance have been described in a number of studies (Butler et al., 2003; Gross, 2014; Richards, 2004; Richards & Gross, 2006; Robinson & Demaree, 2007). In an early study, Richards and Gross (1999) instructed one group of participants to refrain from showing any emotion in response to viewed slides of injured men. Another group was not given any regulatory instructions. Results showed that although the suppressors were successful in maintaining a neutral outward appearance, they performed significantly worse on a memory task of oral information presented alongside the slides. This cost to memory associated with hiding emotional responses has been confirmed in a number of similar studies (Bonanno, Papa, Lalande, Westphal, & Coifman, 2004; Richards & Gross, 2000). Another unintended consequence of suppressing outward emotion was demonstrated in a study in which pairs of participants were asked to watch an upsetting film and then discuss it afterward. In one condition, neither of the pair were given instructions about how to act, whereas in the other condition, one of the pair was asked to hide outward signs of emotion (unbeknownst to their partner). Results showed that the suppressors were deemed as less responsive (i.e., less likely to acknowledge what their partner was saying during the conversation) and slower to respond overall. Partners of suppressors also reported reduced rapport during the conversation, which was linked to suppressors’ deficits in responsiveness (Butler et al., 2003). These cognitive and social costs parallel processes thought to be important in the development and maintenance of social anxiety (Hirsch & Clark, 2004). Individuals engage in self-monitoring in an attempt to manage the impression they are making in social situations (Clark, 2001). This process increases anxiety and has the unintended consequence of reducing social performance as rated by others (Alden & Taylor, 2011) and impairing memory for information present during the social encounter (Daly, Vangelisti, & Lawrence, 1989).

Emotional suppression and self-monitoring have clear emotional, cognitive, and social costs. However, the OG-SD suggests that the motivation for emotion suppression in social contexts may be derived from a belief that authenticity in one’s grief would result in others not being able to cope, casting judgment, or thinking the individual is not normal. If beliefs were held about social networks being compassionate, emotionally receptive, and distress tolerant, we may not see the social fears or associated consequences of social disconnection arise. It is important to make the distinction between the potential to overestimate risk (i.e., “I don’t believe anyone would ever behave kindly if I express my grief”) and self-preservation (i.e., “My network has not been kind in the past or has used my expression of emotion against me”). In a series of qualitative interviews presented by Goodrum (2008), individuals bereaved by homicide described unhelpful responses of their social networks following the individuals’ grief expression. Notable themes were avoidance (e.g., changing the subject or signaling their discomfort through body language), being told “it’s time to move on,” or the potential supporters becoming overwhelmed by their own emotional reaction. These themes were supported in a small cross-sectional study investigating the barriers to grief expression with close friends and family members (Jakoby, 2014). Therefore, future studies that can synthesize bereaved individuals’ experiences of social situations with the experiences of the social network in question will shed light on whether therapeutic interventions targeting social disconnection would be better implemented on the individual level or on the community and societal levels (e.g., the compassionate communities model; Aoun, Breen, White, Rumbold, & Kellehear, 2018).

These results are qualified by a number of limitations. Data were collected online; remote data collection risks unreliable data because it is difficult to ensure participants are not providing low-quality data for financial gain. However, checks of the validity of the data showed no evidence of straight lining or a particularly quick response times for any of the participants. A further limitation is the lack of temporal precedence in the LGCA, which prohibits conclusions regarding causality. Our results are correlational. They are in line with the hypothesis that a reduction in social disconnection may lead to a reduction in psychological distress. However, it is also conceivable that as psychological distress resolves, connectedness to social networks increases because individuals are harboring less difficult emotions to suppress in social situations. Although overall the OG-SD was deemed stable over time, at the factor level, only the Altered Social Self subscale demonstrated good test–retest reliability, which indicates that the other two subscales may be less stable over time. The test–retest sample was relatively small, which means that it is possible that large effects may have been caused within a few individuals. Future research should employ a larger test–retest sample to determine whether the Negative Interpretations of Others’ Reactions to Grief Expression and the Safety in Solitude subscales demonstrate stability over time. Another limitation is the lack of corroborating objective evidence for social disconnection. The OG-SD aims to assess perceived social disconnection, which may be influenced by a negative reporting style, depressed mood, or downstream effects of other symptoms. Future research would benefit from cross-validating scores of perceived social disconnection as measured by the OG-SD with separate reports of social disconnection from friends or family members to parse apart any differences in subjective and objective reporting and to test whether perceived social disconnection predicts poor psychological adaptation over and above objective measures. This would parallel findings in the social support literature (Singh-Manoux, Marmot, & Adler, 2005; Solomon, Mikulincer, & Hobfoll, 1987).

Despite these limitations, this is, to our knowledge, the first study to describe the concept of social disconnection arising after bereavement. The scale describes the antecedents of social disconnection, such as the beliefs precipitating avoidance of social situations or of grief expression, and also describes the consequences of social disconnection, such as the changes to the social self and a sense that being alone is safer than being with other people. By identifying the antecedents and consequences to social disconnection, the scale improves on existing scales and offers clear targets for treatment, such as identifying problematic beliefs. The scale also affords clinicians and researchers the opportunity to measure change in beliefs and consequences linked to perceived social disconnection after bereavement. Our results show that the reductions in social disconnection in the first 12 to 18 months following loss are predictive of reductions in psychological distress during the same period. These results have clinical implications for the treatment of PGD, PTSD, and depression following loss and suggest that a focus on compassion regarding grief expression and communication via self-compassion techniques (Gilbert, 2009), cognitive reappraisal and reclaiming life assignments (Ehlers & Clark, 2000), or systemic approaches (Hayslip & Page, 2013) may be beneficial to grief adaptation.

## Supplemental Material

Smith_Supplemental_Material – Supplemental material for The Masking of Mourning: Social Disconnection After Bereavement and Its Role in Psychological Distress Click here for additional data file. Supplemental material, Smith_Supplemental_Material for The Masking of Mourning: Social Disconnection After Bereavement and Its Role in Psychological Distress by Kirsten V. Smith, Jennifer Wild and Anke Ehlers in Clinical Psychological Science

## Transparency

*Action Editor:* Christopher G. Beevers

*Editor:* Scott O. Lilienfeld

*Author Contributions*

K. V. Smith and A. Ehlers developed the study concept and design, and all of the authors contributed to the questionnaire development. Data collection and statistical analysis were performed by K. V. Smith under the supervision of A. Ehlers. K. V. Smith drafted the manuscript, and A. Ehlers and J. Wild provided critical revisions. All of the authors approved the final manuscript for submission.

## Notes

1. The term *prolonged grief disorder* has been adopted in recent years to reflect the conceptualization of severe and enduring grief (Maercker et al., 2013; Prigerson et al., 2009). However, the majority of clinical and research publications on this condition use the term *complicated grief*. Therefore, to avoid confusion, we use *prolonged grief disorder* to refer to studies that investigated complicated grief using the Inventory of Complicated Grief.

2. Violent loss is defined as resulting from human action or inaction (i.e., suicide, homicide, accident, unintentional overdose, medical negligence) as opposed to illness.

3. Longitudinal scalar measurement invariance was confirmed for the LCFA of psychological distress. For details of invariance testing, the Supplemental Material available online.

4. This analysis was chosen because a latent growth curve of the social symptom composite yielded a significant negative residual variance when combined with the CUFFS model of psychological distress, which resulted in an inadmissible solution that prevented the longitudinal assessment of the composite.

5. A minimum of three time points is recommended for latent growth curve modeling (Duncan & Duncan, 2009), thus longitudinal modeling of the WSAS was not possible.

6. See Supplemental Material for results of the social and occupational functioning hierarchical regressions.
