# Supplementary material for: The Masking of Mourning: Social Disconnection After Bereavement and Its Role in Psychological Distress
Source: Clin Psychol Sci. 2020 May 11;8(3):464–76. doi: 10.1177/2167702620902748 (PMC7252572; doi:10.1177/2167702620902748)
Supplement: Smith_Supplemental_Material – Supplemental material for The Masking of Mourning: Social Disconnection After Bereavement and Its Role in Psychological Distress [file Smith_Supplemental_Material.pdf]

Supplemental Material

Confirming measurement invariance of the longitudinal confirmatory factor analysis of psychological distress

Statistical Analyses.

For longitudinal models, such as the longitudinal factor analysis model (LCFA) of psychological distress, the assumption of measurement invariance can be tested by systematically constraining parameters and comparing model fit of competing models (Kim & Willson, 2014). The first model is an unconstrained model using subdomain indicators (PGD, PTSD and depression) at each time point with autocorrelations among the errors. If model fit is adequate then configural invariance is confirmed, suggesting that the same items (PGD, PTSD and depression) measure the construct of psychological distress across time. Next, factor loadings are fixed to be equal across time to confirm metric invariance, which implies that the meaning of psychological distress does not change over time. Next, mean parameters are fixed across time to confirm scalar invariance (i.e. that participants who have the same value on the latent construct should have equal values on the items the construct is based). Finally, error variances of the indicators are fixed to be equal across time for strict invariance. It has suggested that strict invariance may be impossible to achieve in practice as random errors cannot be consistent at each time point (Wickrama, Lee, O'Neal, & Lorenz, 2016). Therefore, it has been suggested that the assumption of scalar invariance is sufficient to proceed to second-order modelling and interpretation of parameters (Thompson & Green, 2006). The following fit indices determine adequate fit: CFI>.90, TLI>.90, RMSEA<.08, SRMR<.08

(Hu & Bentler, 1999; MacCallum, Browne, & Sugawara, 1996; Wickrama et al., 2016). Measurement invariance was said to be met if changes to fit statistics did not exceed the following CFI (ΔCFI) .01, RMSEA (ΔRMSEA) .015, and SRMR (ΔSRMR) .03 (Chen, 2007; Cheung & Rensvold, 2002)

Results

Measurement model

The results of the nested model comparisons are shown in Table 1. The LCFA of psychological distress with three correlated errors was an excellent fit to the data (CFI = 1.00, TLI = .99, SRMR = .02, RMSEA = .04, χ^2 = 16.17 on df = 12, χ^2:df = 1. 35) confirming configural invariance. The metric model in which factor loadings were constrained to be equal across time did not substantially reduce model fit (ΔCFI = .004, ΔRMSEA = .016, ΔSRMR = .017). The scalar model did not significantly decrease model fit according to the CFI (ΔCFI = .01) and SRMR (ΔSRMR = .03) but was above the threshold for RMSEA (ΔRMSEA = .023). Cheung and Rensvold (2002) suggested that the set of constrained parameters is fundamentally the same across time when the ΔCFI is less than or equal to .01, therefore we can proceed with the understanding that the assumption of scalar measurement invariance is met.

The model for strict invariance was above the threshold for CFI (ΔCFI = .013) and but did not worsen fit according to the RMSEA (ΔRMSEA = .014) and SRMR (ΔSRMR = .010) meaning that overall the assumption of equal random variance across time could not be accepted.

Table 1.

Results from models testing measurement invariance in a longitudinal CFA model of psychological distress

	Model Comparison	CFI (ΔCFI < .01)	RMSEA (ΔRMSEA < .015)	SRMR (ΔSRMR < .03)
Configural LCFA model with autocorrelations (M1)		.979	.101	.073
Configural LCFA with residual errors (M2)		.998	.036	.015
LCFA with metric invariance (M3)	M2 vs M3	.994 (.004)	.052 (.016)	.032 (.017)
LCFA with scalar invariance (M4)	M3 vs M4	.984 (.01)	.075 (.023)	.062 (.03)
LCFA with strict invariance (M5)	M4 vs M5	.971 (.013)	.089 (.014)	.071 (.009)

Note: M = Model. LCFA = Longitudinal Confirmatory Factor Analysis. All of the LCFA models included autocorrelated errors.

Incremental validity.

Social and occupational functioning. Results of the hierarchical regressions using the WSAS as the dependent variable at Time Points 2 and 3 are shown in Table 2. For Time Point 2 at the first step the results indicate the full scales of PGD, $\Delta R^2 = .53$, p <.001; PTSD, $\Delta R^2 = .59$, p <.001; and depression, $\Delta R^2 = .54$ p <.001, all explained significant variance in social and occupational functioning. Importantly, the OG-SD explained a significant amount of additional variance in the WSAS compared to PGD 9%, PTSD 6%, and depression 11%.

A similar pattern of results was observed for Time Point 3. The PGD, $\Delta R^2 = .63$ p <.001; PTSD, $\Delta R^2 = .72$, p <.001; and depression, $\Delta R^2 = .59$, p <.001, symptom scales all explained significant variance in the WSAS at the first step. At the second step the OG-SD explained 6% of additional variance compared to PGD, 3% more than PTSD, and 10% more than depression, all changes were highly significant.

Table 2.

Hierarchical Regressions Predicting Impairment in Social and Occupational Functioning

Time Point	Symptom Scale	Model 1				Model 2						
		DV WASA				DV WASA						
		IV Symptom scale				IV Symptom scale			OG-SD			
		B	SE	β	ΔR^2	B	SE	β	B	SE	β	ΔR^2
2	PGD [a]	.65***	.04	.73	.53	.37***	.06	.41	.16***	.03	.43	.09
	PTSD[b]	.40***	.02	.77	.59	.26***	.03	.50	.14***	.02	.36	.06
	Depression[c]	.99***	.07	.74	.54	.59***	.08	.44	.17***	.02	.44	.11
3	PGD[d]	.73***	.04	.79	.63	.47***	.06	.52	.15***	.02	.37	.06
	PTSD[e]	.46***	.02	.85	.72	.35***	.03	.65	.10***	.02	.26	.03
	Depression[f]	1.03***	.06	.77	.59	.63***	.07	.47	.17***	.02	.44	.10

Note. DV= Dependent Variable, IV = Independent Variable, WASA = Work and Social Adjustment Scale, PGD=PG-13, PTSD=PCL5, Depression = PHQ9.
[a]Model 1: $F\Delta$ (1, 199) = 227.98, p <.001; R^2 = .53. [a]Model 2: $F\Delta$ (1, 198) = 44.09, p <.001; R^2 = .62.
[b]Model 1: $F\Delta$ (1, 199) = 291.24, p <.001; R^2 = .59. [b]Model 2: $F\Delta$ (1, 198) = 31.14, p <.001; R^2 = .65.
[c]Model 1: $F\Delta$ (1, 199) = 234.01, p <.001; R^2 = .54. [c]Model 2: $F\Delta$ (1, 198) = 62.51, p <.001; R^2 = .65.
dModel 1: $F\Delta$ (1, 206) = 351.18, p <.001; R^2 = .63. [d]Model 2: $F\Delta$ (1, 205) = 37.64, p <.001; R^2 = .69.
[e]Model 1: $F\Delta$ (1, 206) = 516.65, p <.001; R^2 = .72. [e]Model 2: $F\Delta$ (1, 205) = 21.25, p <.001; R^2 = .74.
[f]Model 1: $F\Delta$ (1, 206) = 296.63, p <.001; R^2 = .59. [f]Model 2: $F\Delta$ (1, 205) = 66.36, p <.001; R^2 = .69.
All results are significant after alpha correction. *** $p < .001$.

References

Chen, F. F. (2007). Sensitivity of Goodness of Fit Indexes to Lack of Measurement Invariance. *Structural Equation Modeling: A Multidisciplinary Journal, 14*(3), 464-504. doi:10.1080/10705510701301834

Cheung, G. W., & Rensvold, R. B. (2002). Evaluating Goodness-of-Fit Indexes for Testing Measurement Invariance. *Structural Equation Modeling: A Multidisciplinary Journal, 9*(2), 233-255. doi:10.1207/S15328007SEM0902_5

Hu, L., & Bentler, P. M. (1999). Cutoff criteria for fit indexes in covariance structure analysis: Conventional criteria versus new alternatives. *Structural Equation Modeling: A Multidisciplinary Journal, 6*(1), 1-55. doi:10.1080/10705519909540118

Kim, E. S., & Willson, V. L. (2014). Measurement invariance across groups in latent growth modeling. *Structural Equation Modeling: A Multidisciplinary Journal, 21*(3), 408-424.

MacCallum, R. C., Browne, M. W., & Sugawara, H. M. (1996). Power analysis and determination of sample size for covariance structure modeling. *Psychological Methods, 1*(2), 130.

Thompson, M. S., & Green, S. B. (2006). Evaluating between-group differences in latent variable means. In G. R. Hancock & R. O. Mueller (Eds.), *Structural equation modeling: A second course* (pp. 119-169). Greenwich: CT: Information Age.

Wickrama, K. K., Lee, T. K., O'Neal, C. W., & Lorenz, F. O. (2016). *Higher-order growth curves and mixture modeling with Mplus: A practical guide*. New York, USA: Routledge.
